# Rekonstruktion des vorderen Kreuzbands mit der Quadrizepssehne und rechteckigem femoralen Knochenkanal

**DOI:** 10.1007/s00113-025-01547-0

**Published:** 2025-03-05

**Authors:** Christian Fink, Andrea Marchetti, Tobias Schwäblein, Mirco Herbort, Elisabeth Abermann

**Affiliations:** 1 Gelenkpunkt – Sport und Gelenkchirurgie, Olympiastraße 39, 6020 Innsbruck, Österreich; 2 Klinik für Orthopädie und Traumatologie Universitätsklinikum, Triest, Italien; 3 Klinik für Unfall- und Wiederherstellungschirurgie, BG Klinikum Bergmannstrost, Halle (Saale), Deutschland; 4 Research Unit für Sportmedizin des Bewegungsapparates und Verletzungsprävention, UMIT, Hall, Österreich; 5 OCM Klinik, München, Deutschland

## Einleitung

Die Diskussion über die Rekonstruktion des vorderen Kreuzbandes (VKB) mit einem oder zwei Bündeln hat zu einer produktiven Forschungsperiode und einem tieferen Verständnis der Anatomie und Biomechanik des nativen VKB geführt [1]. Jüngste Erkenntnisse deuten darauf hin, dass der intraligamentäre Teil des nativen VKB eher einem „Band“ ähnelt (ca. 11–16 mm breit und 2,5–3,4 mm dick), und dass das arthroskopisch beobachtete „Doppelbündelerscheinungsbild“ durch eine Verdrehung dieser flachen Struktur entsteht [2, 3].

**Abb. 1:**
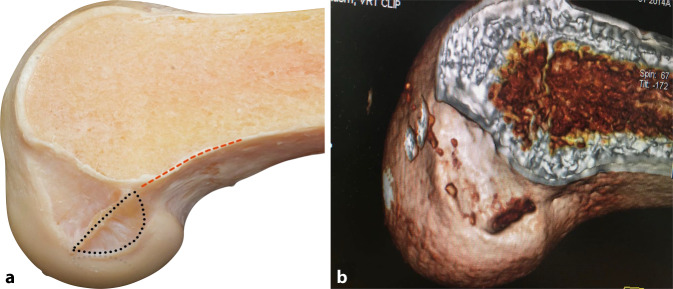
**a** Anatomisches Präparat mit direkter VKB-Insertion femoral in Verlängerung der dorsalen Kortikalis *(rot strichlierte Linie*) und indirektem Insertionsareal (*schwarz punktiert*). **b** Postoperative 3D-CT-Rekonstruktion einer VKB-Rekonstruktion mit flachem femoralen Kanal im anatomischen Insertionsareal

Der native femorale Ursprung hat eine schmale „direkte“ Komponente, die in Kontinuität von der posterioren femoralen Kortikalis ausgeht und sich entlang des lateralen interkondylären Kamms erstreckt, sowie fächerartige „indirekte“ Fasern, die sich zum posterioren chondralen Rand des femoralen Kondylus erstrecken ([2]; Abb. 1a). Rechteckige Knochentunnel können deshalb die native Anatomie möglicherweise besser nachbilden und ermöglichen eine Transplantatrotation während der Kniebeugung, die der Biomechanik des nativen vorderen Kreuzbandes ähnelt (Abb. 1b).

Sowohl Patellarsehnen- als auch Quadrizepssehnen(QS)-Transplantate ähneln morphologisch eher dem bandförmigen VKB. Allerdings ist insbesondere die Entnahme von QS-Transplantaten technisch anspruchsvoll und der für die offene Entnahme verwendete Längsschnitt kosmetisch weniger vorteilhaft als die Entnahme der Hamstring-Sehne.

Daher werden bei der dargestellten Operationstechnik [4] folgende Ziele verfolgt:

1. Optimierung der femoralen Tunnelform, um eine anatomiegerechtere Transplantatausrichtung und Biomechanik zu erreichen,
2. Standardisierung der QS-Entnahme, um technische Schwierigkeiten und Morbidität zu reduzieren.

Aufklärung: Neben den allgemeineinen Operationsrisiken (z. B. Thrombose, Infektion, Gefäß- und Nervenverletzungen) ist v. a. auf mögliche postoperative Bewegungseinschränkungen und ein bestehendes Wiederverletzungsrisiko hinzuweisen. Außerdem sollte auf die Wichtigkeit einer zielgerichteten, langwierigen physikalischen Therapie hingewiesen werden. Natürlich ist im Aufklärungsgespräch auf alternative Therapieoptionen einzugehen.

## Operationstechnik

### Positionierung

**Abb. 2:**
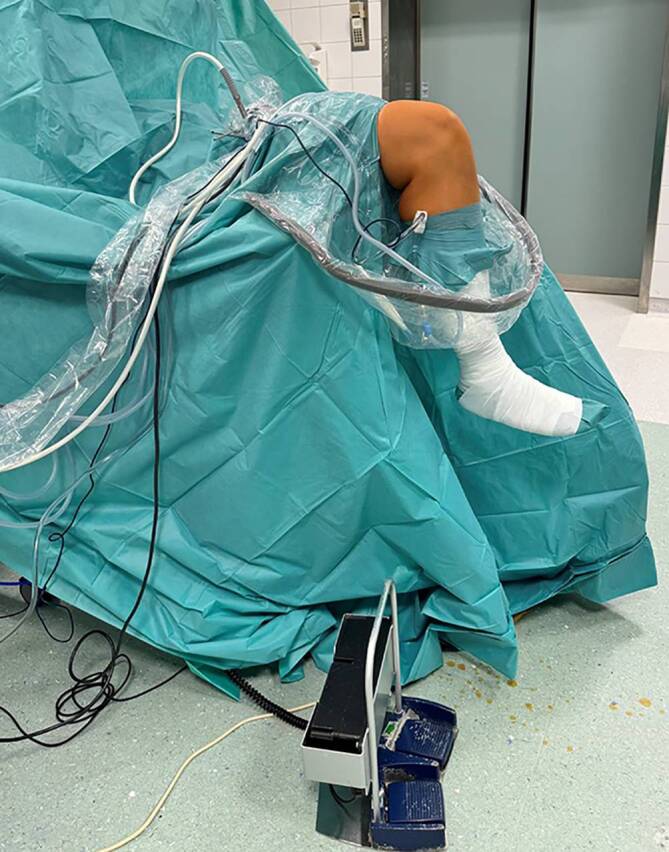
Lagerung mit elektrischem Beinhalter im OP, der eine Beweglichkeit zwischen 0° und 120° zulässt und mittels Fußschalter vom Operateur bedient werden kann

Der Patient wird mit einer Oberschenkelblutsperre in Rückenlage positioniert. Die Positionierung muss eine Kniebewegung zwischen 0 und 120 Grad ermöglichen. Wir bevorzugen die Verwendung eines elektrischen Beinhalters (Fa. Maquet, Rastatt, Deutschland, Abb. 2).

### Transplantatentnahme

**Abb. 3:**
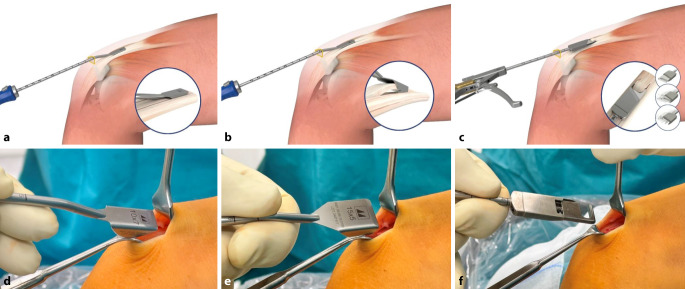
Entnahmeinstrumentarium für die Quadrizepssehne (Fa. Medacta International, Castel San Pietro, Schweiz): **a,d** Doppelmesser, **b,e** Sehnenseparator, **c,f** Sehnenschneider

**Abb. 4:**
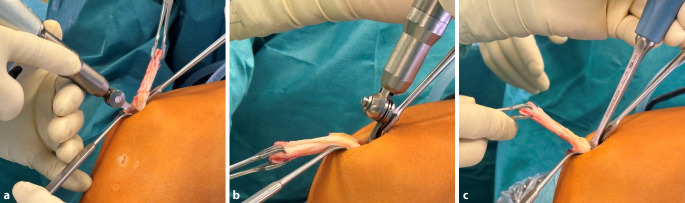
Knochenblockentnahme: **a** Mit einer oszillierenden Säge wird mithilfe eines kleinen Sägeblatts primär die Dimension des Knochenblocks an der Patellavorderfläche festgelegt. **b** Anschließend wird die Tiefe ebenfalls mit der Säge definiert (ca. 5 mm). **c** Der Knochenblock wird schließlich mit einem Osteotom gehoben

**Abb. 5:**
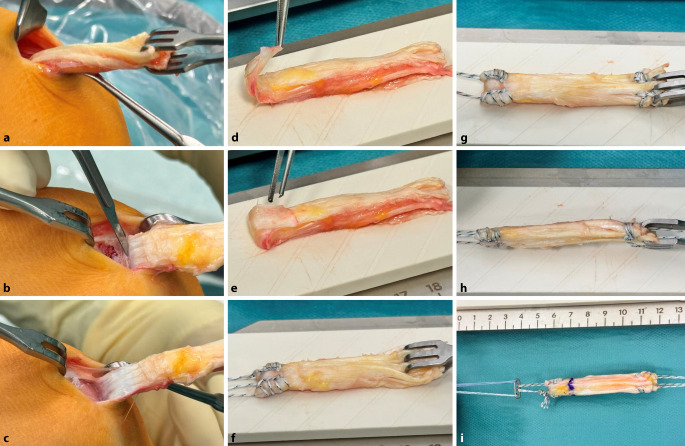
Transplantatentnahme mit Periostlappen: **a** Das proximale sehnige Ende des Transplantats wird mit einer Sehnenfasszange gegriffen. **b,c** Unter Zug an der Sehne wird mit dem Messer scharf nach distal präpariert (**b**), bis das Periost schließlich „abgeschält“ werden kann (**c**). **d–f** Der Periostlappen wird dann über den Sehnenanteil umgeschlagen (**d**, **e**) und mit nichtresorbierbarem Fadenmaterial in Krackow-Technik angeschlungen (**f**). **g**, **h** Nachdem auch das distale Sehnenende angeschlungen wurde, ist die Bandform gut zu erkennen. **i** Fertig geknüpftes Transplantat mit Einzugsfäden

**Abb. 6:**
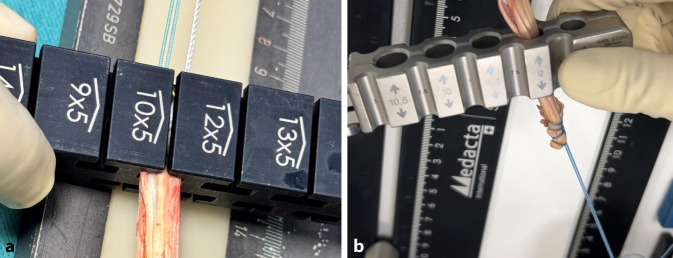
Größenbestimmung: **a** flache Größenbestimmung des proximalen Transplantatendes, **b** runde Größenbestimmung des distalen Sehnenendes

- Bei einer Kniebeugung von 90**°** wird ein 2,5 –3 cm langer transversaler Hautschnitt über dem oberen Rand der Patella vorgenommen.
- Ein langer Langenbeck-Retraktor wird eingeführt und eine Inzision in Bursa und Paratenon vorgenommen, um die QS subkutan freizulegen.
- Ein Doppelmesser (Fa. Medacta International, Castel San Pietro, Schweiz) mit einer Tiefe von 7 mm und der gewählten Breite von 8–14 mm (je nach Körpergröße des Patienten) wird dann eingeführt (Abb. 3a,d). Das Instrument wird nach proximal vorgeschoben, wobei der Mittelpunkt des Doppelmessers leicht seitlich vom Mittelpunkt des oberen Randes der Patella positioniert wird, um den längsten Sehnenspiegel der QS zu erfassen. Das Doppelmesser wird bis zu einer Mindesttiefe von 70 mm eingeführt (Abb. 3a,d)
- Die Dicke des Transplantats in der koronalen Ebene wird mit einem Separator (Fa. Medacta International, Castel San Pietro, Schweiz) bestimmt (Abb. 3b,e). Der Separator hat eine stumpfe Referenzseite, die auf dem oberen Teil der Sehne aufliegt, und eine scharfe Schneideklinge in einer Tiefe von 5 mm, die die Sehne in der koronalen Ebene einschneidet. Er wird nach proximal bis zur gleichen Länge eingeführt. Ein 10 × 5 mm Transplantat entspricht etwa einem runden 8 mm, ein 12 × 5 Transplantat einem runden Transplantat mit Durchmesser 9 mm (Abb. 6).
- Mit einem speziellen Sehnenschneider (Fa. Medacta International, Castel San Pietro, Schweiz) wird der Sehnenstreifen subkutan auf die gewünschte Länge abgeschnitten und das sehnige Ende des Transplantats entnommen. (Abb. 3c,f).
  Die QS kann mit und ohne Knochenblock entnommen werden. Vor allem in der Revisionssituationen kann der Knochenblock einen Vorteil bieten.
- Bei Verwendung der Knochenblocktechnik wird eine oszillierende Säge mit kleinem Sägeblatt verwendet, um aufeinanderfolgende sagittale, axiale und koronale Schnitte in der Patella in einer Tiefe von 5 mm zu machen (Abb. 4a–c). Die endgültige koronale Trennung des Knochenblocks erfolgt mit einem Osteotom, um ein besseres taktiles Feedback und eine bessere Kontrolle zu erhalten.
- Wenn die QS ohne Knochenblock entnommen wird, wird ein Streifen Periost von der vorderen Patella durch schrittweise subperiostale Dissektion entnommen, wobei das Transplantat unter Spannung zurückgeschält wird (Abb. 5a–c)
- Zum Schließen des Defekts wird der Langenbeck-Retraktor erneut eingeführt. Der Sehnendefekt wird mit resorbierbaren Nähten (0 Vicryl, Fa. Ethicon) in fortlaufender Nahttechnik verschlossen. Eine Nadel mit kurzem Radius wird für die Manövrierfähigkeit verwendet. In den meisten Fällen erfolgt die Sehnenentnahme in Teilschichttechnik. Um das Austreten von Arthroskopieflüssigkeit während des Eingriffs zu minimieren, wird nach dem Schließen der Sehne oberflächlich eine Kompresse zwischen Sehne und Haut eingeführt. Die Haut wird am Ende des Eingriffs geschlossen.

### Transplantatvorbereitung

1. Das proximale (sehnige) Ende des Transplantats, das zum tibialen Ende des implantierten Transplantats wird, wird mit einer Krackow-Nahttechnik mit 2 nichtresorbierbaren Fäden der Stärke 2 (Powerknot, Fa. Medacta International, Castel San Pietro, Schweiz) angeschlungen (Abb. 5g–i).
2. Der Knochenblock wird so zugeschnitten, dass er in die gewählte rechteckige Transplantatschablone (4/5 mm × 8, 9 oder 10 mm; Fa. Medacta) passt. Anschließend werden 2 leicht versetzte 1,5-mm-Löcher in den Block gebohrt, und zwar an der Verbindung zwischen dem distalen und dem mittleren sowie dem mittleren und dem proximalen Drittel des Blocks. Ein nichtresorbierbarer Faden der Stärke 2 (Powerknot, Fa. Medacta International, Castel San Pietro, Schweiz) wird durch den Block und die mittleren beiden Löcher des femoralen Fixierungsbuttons geschlungen. In Revisionsfällen wird ein Knochenblocktransplantat einem Periostlappentransplantat vorgezogen.
  Wenn die Quadrizepssehne als reines Weichteiltransplantat entnommen wurde, wird das Periost zum Transplantat hin zurückgefaltet, um eine glatte Vorderkante zu bilden, und der Lappen wird mit 2 nichtresorbierbaren Fäden der Stärke 2 (Powerknot, Fa. Medacta) in die Krackow-Nahttechnik eingearbeitet (Abb. 5d–f).
3. Das nichtresorbierbare Nahtmaterial (Nr. 2 Powerknot, Fa. Medacta) vom Knochenblock/Periostende des Transplantats wird in der für den femoralen Knochentunnel geeigneten Länge an den Fixierungsbutton geknüpft (Abb. 5h). Die ideale Länge ist so bemessen, dass der Knochenblock bündig oder leicht vertieft in der femoralen Tunnelöffnung liegt. Wenn ohne Knochenblock gearbeitet wird sollten mindestens 15 mm des Transplantats im Tunnel liegen (Abb. 6).

#### Femoraler Tunnel

**Abb. 7:**
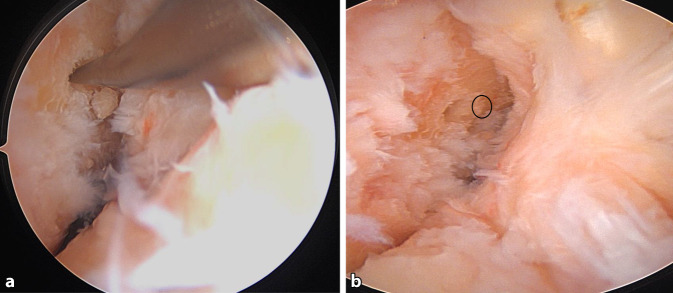
Femorale Tunnelplatzierung: **a** Markierung der femoralen Position mit einer Mikrofrakturale. **b** angelegtes Markierungsloch für den femoralen Markierungsdraht (*schwarzer Kreis*)

**Abb. 8:**
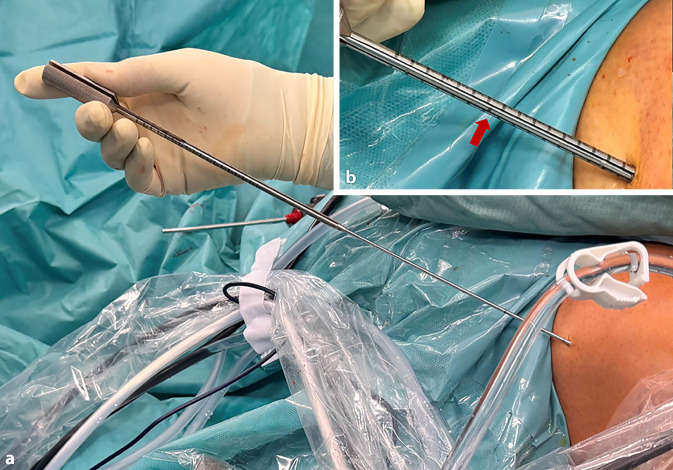
Längenmessung des femoralen Kanals von extraartikulär mithilfe einer Lasermarkierung am Führungsdraht (*roter Pfeil*) und dazu passendem Messgerät (Fa. Medacta)

**Abb. 9:**
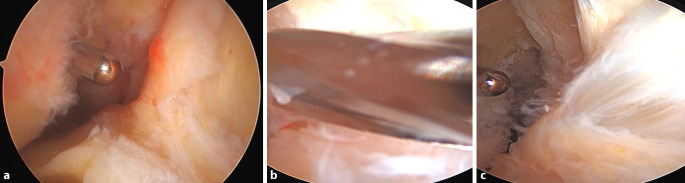
Überbohren des Markierungsdrahts (**a**) mit einem 4,5 mm Bohrer (**b**), Kirschner-Draht im 4,5 mm Bohrloch (**c**)

**Abb. 10:**
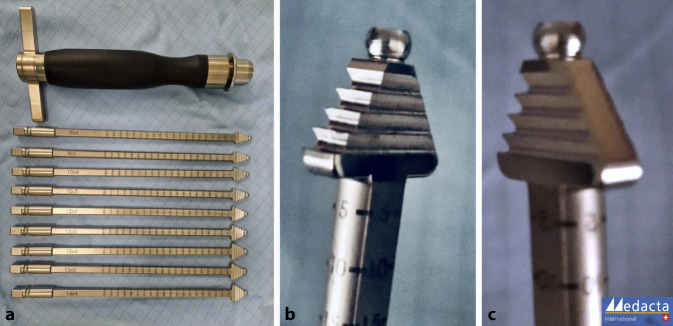
Raspel-Set für die Anlage eines flachen femoralen Kreuzbandkanals. (Fa. Medacta International, Castel San Pietro, Schweiz; mit freundlicher Genehmigung)

1. Nach der routinemäßigen diagnostischen Arthroskopie werden die gerissenen VKB-Anteile reseziert, wobei aber der tibiale Bandrest, wenn möglich, erhalten wird.
2. Bei einer Kniebeugung von 120° wird ein 2,4 mm Führungsdraht durch das mediale Portal in die native femorale VKB-Insertion gebohrt, bis die Markierung auf dem Draht bündig mit der Femurkondyle abschließt. Der Eintrittspunkt für den Führungsdraht liegt leicht proximal und posterior zum Mittelpunkt des direkten Ursprungs des ACL (Abb. 7). Die Position wird von beiden Portalen aus überprüft.
3. Die Tunnellänge wird extraartikulär über den Führungsdraht gemessen, wobei die laterale Femurkortikalis und die Lasermarkierung auf dem Draht als Referenz dienen (Abb. 8).
4. Der Führungsdraht wird bikortikal mit einem 4,5-mm-Bohrer aufgebohrt (Abb. 9).
5. Die rechteckige Raspel (Fa. Medacta International, Castel San Pietro, Schweiz) mit dem passenden Transplantatdimensionen (10 × 4 mm, 10 × 5 mm, 12 × 4 mm und 12 × 5 mm sind am häufigsten) wird über den Führungsdraht intraartikulär eingeführt. Bei einer Kniebeugung von 120° sollte die Raspel horizontal ausgerichtet sein, wobei die glatte Seite zum HKB zeigt (Abb. 10, 11 und 12). Der Tunnel wird bis zu einer Tiefe von 25–30 mm geraspelt. Dies ist ca. 10 mm tiefer als die Länge des Knochenblocks oder 10 mm mehr als die Transplantatlänge, die in den Kanal eingezogen werden soll, um Platz für das Kippen des femoralen Fixierungsbuttons zu schaffen.

**Abb. 11:**
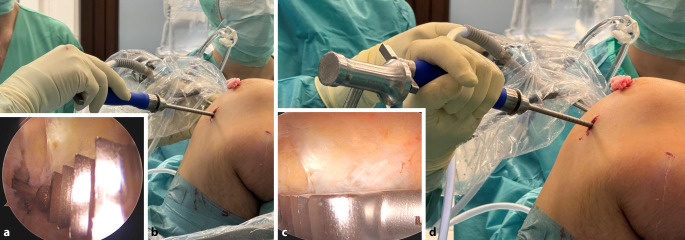
Anlage des flachen femoralen Kanals. **a, b, c** Einbringen der Raspel mit der flachen Seite zum hinteren Kreuzband und Aufsetzen auf den Zieldraht. **d** Einschlagen der Raspel mit dem Hammer in 110°-flektiertes Kniegelenk

**Abb. 12:**
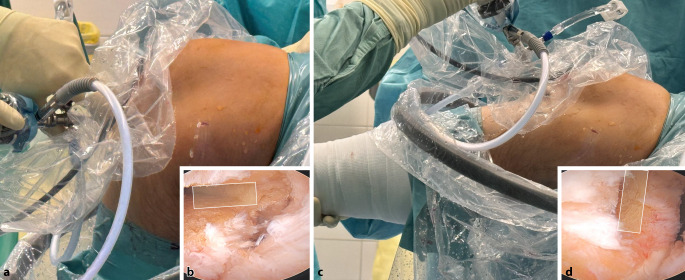
Flacher femoraler Kanal (*Rechtecke* markieren den femoralen Ausgang des Knochenkanals) bei ca. 100°-gebeugtem Kniegelenk (**a, b**; annähernd *horizontal*) und streckungsnah (**c, d**; annähernd *vertikal*)

#### Tibialer Tunnel

**Abb. 13:**
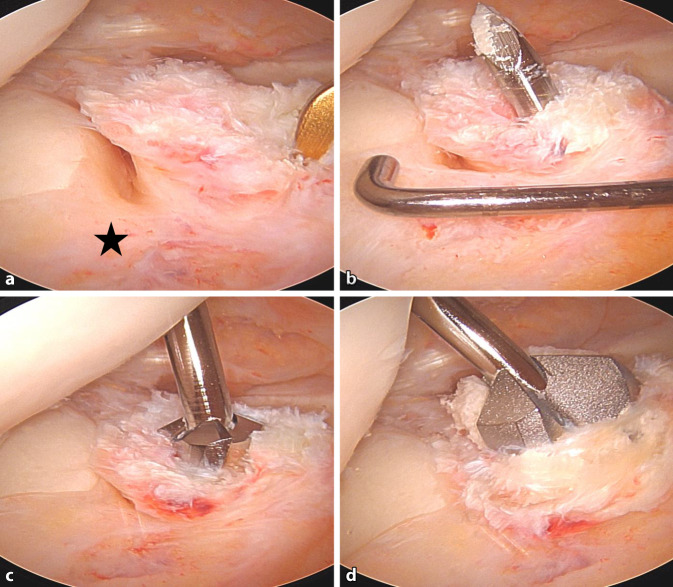
Tibiale Kanalanlage: **a** Einbringen des tibialen Kreuzbandzielgeräts in den Bandstumpf auf Höhe des lateralen Meniskusvorderhorns (*Stern*). **b** Führungsdraht im Bandstumpf. **c**, **d** Schrittweises Aufbohren auf die passende Dimension

Der tibiale Zielbügel (Fa. Medacta International, Castel San Pietro, Schweiz) wird durch das mediale Portal eingeführt und in Bezug auf das Vorderhorn des lateralen Meniskus und den VKB-Stumpf platziert (Abb. 13a). Der 2,4-mm-Führungsdraht wird mit dem Zielgerät an den definierten Insertionspunkt eingebracht (Abb. 13b). Anschließend wird das Knie gestreckt, um die Tunnelplatzierung bezüglich eines etwaigen Impingement zu überprüfen. Nun wird der Führungsdraht mit dem gewünschten Bohrerdurchmesser überbohrt (Abb. 13c, d).

### Transplantateinführung

**Abb. 14:**
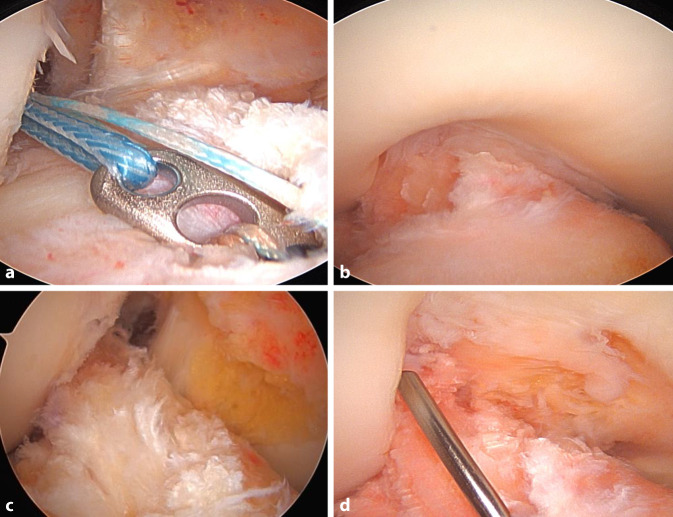
Transplantateinzug: **a** Fixationsbutton intraartikulär beim Kipptest. **b,c** Eingezogenes Transplantat in voller Streckung (**b**) und in 90° gebeugtem Knie (**c**). **d** Führungsdraht für die Interferenzschraube unter arthroskopischer Sicht lateral des Transplantats

**Abb. 15:**
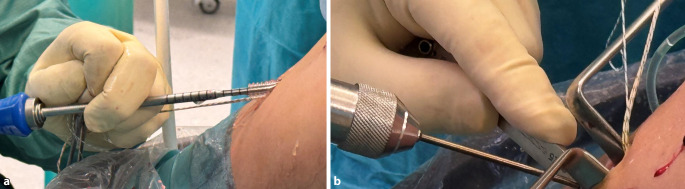
Tibiale Transplantatfixation: **a** Einbringen der Biointerferenzschraube (Fa. Medacta) über den Führungsdraht bei ca. 20° gebeugtem Kniegelenk. **b** Back-up-Fixation: Knüpfen der Fäden über eine Knochenbrücke durch ein 2,5 mm Bohrloch ca. 5 mm distal des tibialen Kanals

**Abb. 16:**
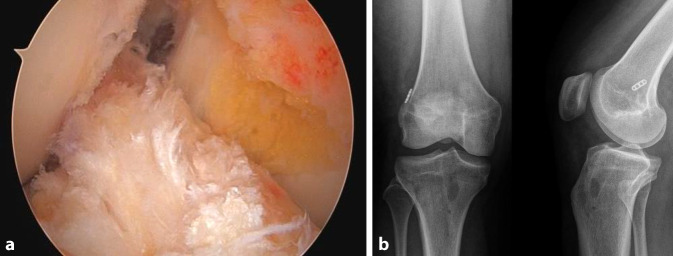
Postoperative Kontrolle: **a** arthroskopische Dokumentation des Transplantats, **b** Röntgenaufnahme in 2 Ebenen

1. Ein Bankart-Stift wird über das mediale Portal eingeführt, um einen Vicrylfaden durch den femoralen Tunnel zu führen. Die Fadenschlinge wird intraartikulär über den tibialen Tunnel gefasst und extraartikulär nach distal gezogen. Die Schlinge wird dann verwendet, um die Führungsfäden für den Fixationsbutton (Fa. Medacta International, Castel San Pietro, Schweiz) durch das Knie und durch die Femurtunnelöffnung nach proximal-lateral herauszuziehen.
2. Das Transplantat wird durch Ziehen an den Führungsfäden eingeführt. Die korrekte Ausrichtung im Tibiatunnel ist so, dass die Oberfläche des Spongiosablocks nach anterior zeigt.
3. Das Umklappen des Fixationsbuttons (Fa. Medacta International, Castel San Pietro, Schweiz) wird unter arthroskopischer Sicht bestätigt, um sicherzustellen, dass sich die Führungsfäden in korrekter Position befindet (Abb. 14a).
4. Sobald das Transplantat aus dem proximalen tibialen Tunnel in den Gelenkspalt austritt, wird der Knochenblock vor dem Einführen in den femoralen Tunnel mithilfe eines arthroskopischen Palpationshäkchens intraartikulär geführt und korrekt gedreht. Bei rechten Knien ist eine Drehung im Uhrzeigersinn erforderlich, bei linken eine Drehung gegen den Uhrzeigersinn. Dieser Schritt lässt sich leichter durchführen, wenn sich das Knie in Streckung befindet, da das Transplantat weniger gedreht werden muss, je näher das Knie der vollständigen Streckung ist.
5. Wenn der Knochenblock richtig gedreht ist, wird das Knie wieder auf 90° gebeugt und das Transplantat vollständig in den rechteckigen Knochentunnel gezogen, bis der Fixationsbutton (Fa. Medacta International, Castel San Pietro, Schweiz) kippt. Dies wird erreicht, indem zunächst am vorderen Ende des Buttons gezogen wird, bis auch das hintere Ende den lateralen kortikalen Knochen passiert hat. In der Regel ist eine spürbare Verringerung des Widerstands zu verzeichnen, wenn das hintere Ende des Fixationsbuttons den proximalen Tunnel passiert.
6. Mit Spannung auf den distalen Nähten des Transplantats wird das Knie ca. 10-mal von 0–90**°** bewegt, um das Transplantat zu konditionieren (Abb. 14b, c).
7. Distal wird eine Hybridfixierung verwendet. Eine vollständig mit Gewinde versehene kanülierte bioresorbierbare Interferenzschraube (Bio-Interferenzschraube, Vollgewinde, Fa. Medacta International, Castel San Pietro, Schweiz), die dem Tunneldurchmesser entspricht und in der Regel 25 mm lang ist, wird über einen Führungsdraht lateral zum Transplantat eingeführt (Abb. 15a), um eine anteromediale Kompression des Transplantats zu erreichen. Die Position der Schraube wird arthroskopisch überprüft (Abb. 14d), um ein intraartikuläres Hervortreten zu vermeiden, bevor die Nahtenden über eine kortikale Knochenbrücke geknüpft werden. Für die Knochenbrücke wird ein 2,5 mm großes Bohrloch ca. 3–5 mm unterhalb des tibialen Kanals angelegt (Abb. 15b), und 2 der 4 distalen Powerknot-Fäden (Fa. Medacta International, Castel San Pietro, Schweiz) werden von außen nach innen durch das neue Bohrloch geführt. Die Fäden werden mit den verbleibenden distalen Powerknot-Fäden verknüpft, sodass der endgültige Knoten im Tibiatunnel liegt und subkutan nicht tastbar ist.
8. Die endgültige Position und Spannung des Transplantats werden arthroskopisch mithilfe eines arthroskopischen Tasthäkchens kontrolliert und fotodokumentiert (Abb. 16a). Postoperativ erfolgt eine Röntgenkontrolle in 2 Ebenen (Abb. 16b).

## Postoperative Versorgung

Es wird regelhaft eine Knieschiene verwendet, die die Beugung auf 90° begrenzt. Initial wird v. a. Wert auf eine gute Aktivierung der Quadrizepsmuskulatur und die Forcierung der Extension gelegt. Außerdem sollte auf eine frühzeitige Reduktion der Schwellung durch Lymphdrainagen sowie Kälte- und Kompressionsanwendungen geachtet werden. Die Patienten werden in der Regel 2 Wochen lang mit teilweiser Gewichtsbelastung (ca. 20 kg) an Unterarmstützkrücken mobilisiert. Danach wird mit voller Gewichtsbelastung und freiem Bewegungsumfang begonnen. Es wird empfohlen, mindestens 8 bis 12 Wochen lang 2‑ bis 3‑mal/Woche Physiotherapie durchzuführen. Bei Zusatzpathologien (z. B. Meniskusrissen oder Knorpelverletzungen) muss das Nachbehandlungsprogramm bezüglich Belastung- und Bewegungssteigerung entsprechend adaptiert werden.
